# Improvement in mild anti-IgLON5 encephalopathy without immunotherapy: a case report

**DOI:** 10.1186/s12883-021-02145-4

**Published:** 2021-03-17

**Authors:** Yuting Wang, Xiuling Wu, Baoquan Lu

**Affiliations:** Department of Neurology, Tangshan Gongren Hospital, 27 Wenhua Road, Tangshan, 063000 Hebei People’s Republic of China

**Keywords:** Anti-IgLON5 encephalopathy, Immunotherapy, Infection, Antiviral, Case report

## Abstract

**Background:**

Anti-IgLON5 antibody-related encephalopathy is a recently discovered and rare autoimmune disease, and its diagnosis and treatment are more challenging than for other autoimmune encephalopathic diseases. Sleep disorder is the most prominent symptom of the disease. It can also present with gait instability, dysarthria, dysphagia, dementia, ataxia, autonomic nervous system dysfunction, chorea, vertical gaze paralysis, and other symptoms. Immunotherapy remains the primary treatment for this disease; however, there is no definitive conclusion regarding the effect of immunotherapy. The clinical symptoms of the reported cases of anti-IgLON5 antibody-related encephalopathy were generally severe. However, the symptoms in our patient were mild and relieved without immunotherapy, unlike the previously reported cases.

**Case presentation:**

A 62-year-old man presented with behavioural abnormalities and involuntary movements after nearly 2 months of fever and headache. He also had symptoms of mild sleep disorder. Due to the abnormal levels of infection-related indicators, antiviral treatment was started on the day of admission. The serum analysis confirmed the presence of IgLON5 antibody, and the patient was found to be genetically susceptible. The patient’s symptoms resolved rapidly without immunotherapy and did not recur.

**Conclusions:**

This case demonstrated that IgLON5 antibody-related encephalopathy might have mild manifestations. Infection and a genetic predisposition may be important causes for the disease. Patients with a mild disease may have a better prognosis.

## Background

Anti-IgLON5 antibody-related encephalopathy is an exceedingly rare autoimmune disease of the central nervous system. The number of cases reported worldwide is limited, and all reported cases had severe clinical symptoms requiring immunotherapy. We report a case of mild anti-IgLON5 antibody-related encephalopathy and its remission without the use of immunotherapy. This case is an unusual presentation of the disease.

## Case presentation

A 62-year-old man presented with a 2-month history of intermittent fever and persistent headache. His medical history was unremarkable. According to his wife, he occasionally exhibited increased movement and vocalised during sleep. He was treated at a local hospital and showed no improvement. He was transferred to our neurology clinic for the treatment of worsening behavioural abnormalities such as unintelligible speech, incorrect answers to questions, inability to communicate with others, failure to recognise his family members, and irritability. The patient also had an episode of urination into his slippers 1 day before admission.

On initial examination, the patient showed aggressive behaviour and agitation, which was mainly verbal, such as swearing. He did not indulge in any obvious physical attack but refused to be touched. He urinated once every 30 min, and the volume of urine each time was approximately 50 mL. Physical examination 1 day after admission: the patient had cognitive decline, characterised by poor orientation, calculation, and memory, and could only answer simple questions such as his name, and he refused to eat. His temperature was 38.2 °C. Cranial nerve examination showed no abnormality, the muscle strength of the limbs was normal, and the pathological signs were negative. During the physical examination and communication with the patient, involuntary movements of both upper limbs were observed.

Routine laboratory tests results showed a C-reactive protein level of 13.15 mg/L (normal, 0–5 mg/L), erythrocyte sedimentation rate of 40 mm/h (normal, 0–20 mm/h), lymphocyte ratio of 0.12 (normal, 0.20–0.40), and procalcitonin level of 0.19 ng/mL (normal, 0–0.05 ng/mL). Routine urinalysis and computed tomography of the lung showed no abnormalities. Electroencephalography showed a basic rhythm of 8 cycles/s and poor alpha wave formation in the right occipital area. Results of magnetic resonance imaging of the brain were unremarkable.

Based on the symptoms such as fever, sudden behavioural abnormalities, and abnormal indices indicative of infection, we considered the possibility of viral encephalitis and initiated antiviral therapy with acyclovir 0.5 g every 8 h intravenously guttae for 19 days (20 mg/kg/d) on the day of admission. The behavioural abnormalities ceased 3 days later. During the first 2 days of admission, the patient exhibited involuntary and abnormal movements similar to actions such as knitting, hitting, and picking up or sorting objects. These movements were seen during sleep, wakefulness, and even when communicating with others.

**Fig. 1:**
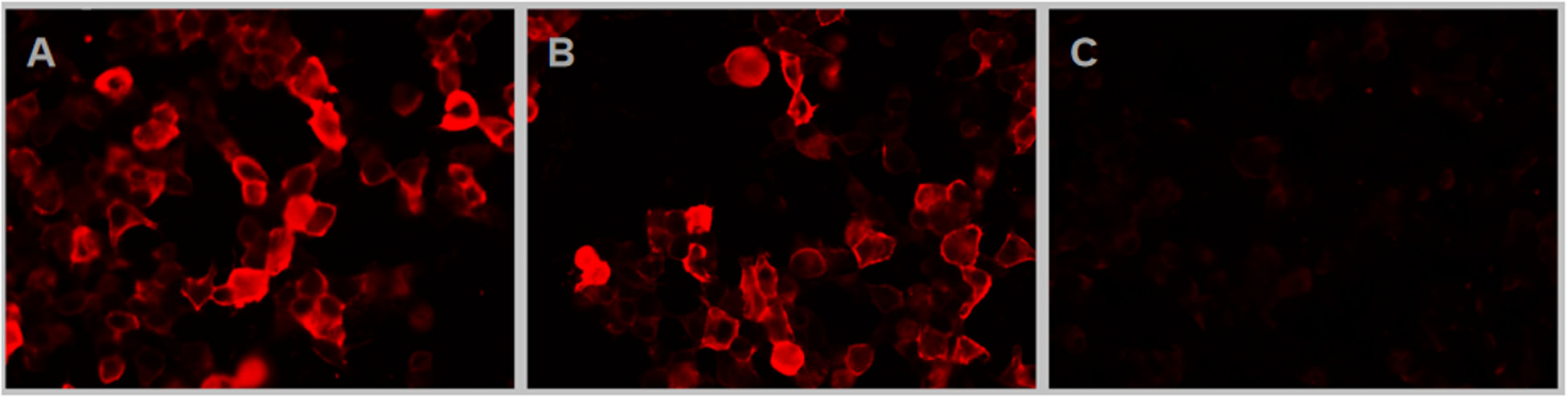
IgLON5 antibody cell-based assay. ^a^ Supplement: **a** IgLON5 IgG antibodies 1:30 in the serum. **b** IgLON5 IgG antibodies 1:10 in the serum. **c** IgLON5 IgG antibodies negative in the CSF

On day 6, lumbar puncture results showed cerebrospinal fluid (CSF) pressure of 326 mm H_2_O (normal, 80–180 mm H_2_O). CSF examination revealed an elevated protein level (0.96 g/L; normal, 0.12–0.6 g/L) and leukocyte counts (34/μL; normal, ≤5/μL) with monocytic predominance. Due to the atypical symptoms, the presence of autoimmune encephalitis-related antibodies was investigated. Autoantibody screening using an in-house assay (KingMed Diagnostics Reference Laboratory, Guangzhou, Guangdong, China) showed the presence of IgLON5 IgG antibodies (1:30) in the serum but not in the CSF. Cell-based assay was used to detect the anti-IgLON5 antibodies, as shown in Fig. 1. IgG4 and IgM were further identified by cell-based methods to determine the IgG antibody subclasses. Paraneoplastic antibodies were not present. Viral antibody testing was negative on two occasions (including enterovirus EV nucleic acid, adenovirus nucleic acid, cytomegalovirus nucleic acid, herpes simplex virus type I nucleic acid, herpes simplex virus type II nucleic acid, EB virus nucleic acid, enterovirus 71 nucleic acid, Coxsackie virus nucleic acid, Japanese encephalitis virus nucleic acid, varicella herpesvirus nucleic acid, rubella virus nucleic acid). With the administration of antiviral therapy alone (i.e., without immunotherapy), his sleep normalised, and his condition steadily improved.

**Fig. 2:**
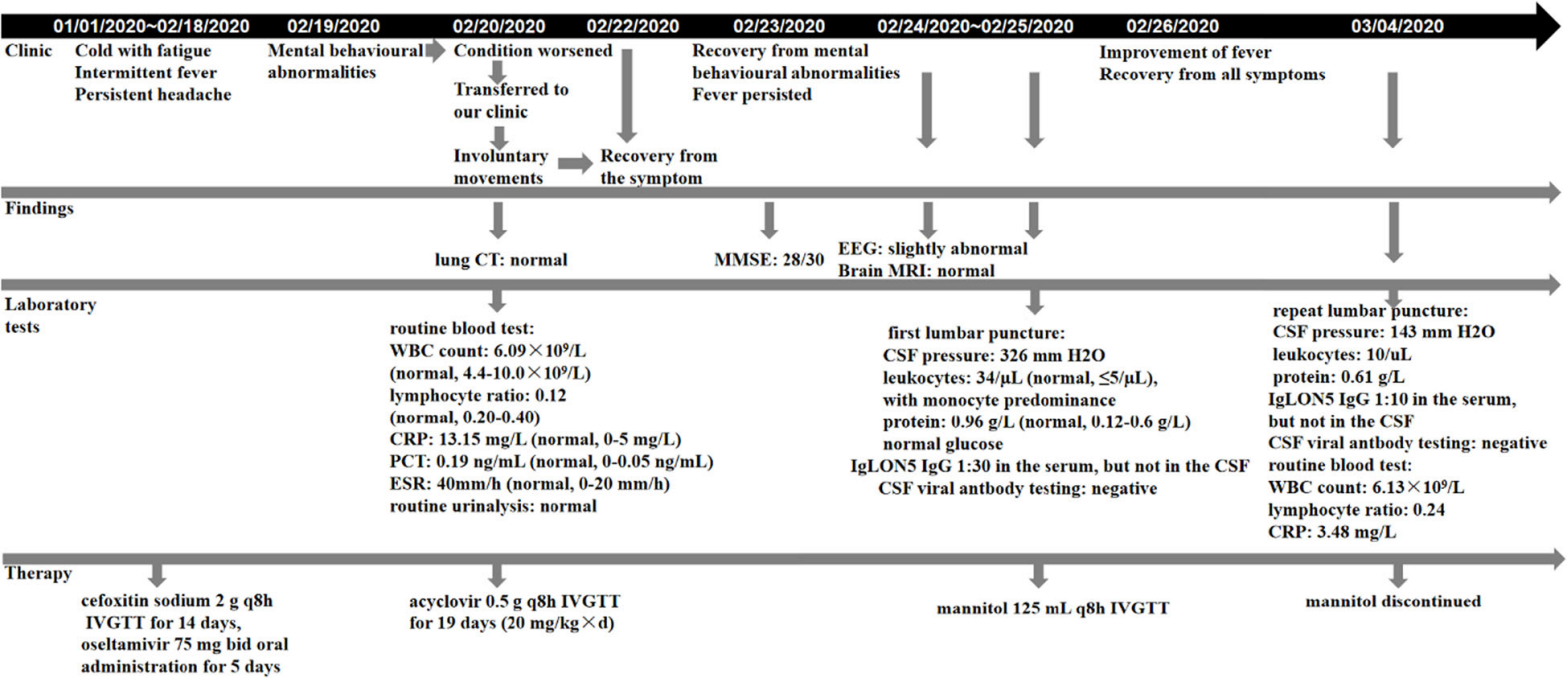
Timeline of symptoms. ^a^ Supplement: On 27 March 2020, HLA typing results revealed HLA-DQB1*05:01 and HLA-DRB1*10:01 alleles. CT: computed tomography; MMSE: Mini-Mental State Examination; EEG: electroencephalography; MRI: magnetic resonance imaging; WBC: white blood cell count; CSF: cerebrospinal fluid; CRP: C-reactive protein; PCT: procalcitonin; ESR: erythrocyte sedimentation rate; IVGTT: intravenously guttae

A repeat lumbar puncture on day 14 indicated a normal CSF pressure of 143 mm H_2_O. CSF parameters had also improved, with a protein level of 0.61 g/L and a leukocyte count of 10/μL. The serum anti-IgLON5 antibody titre decreased to 1:10 and it was negative in the CSF, as shown in Fig. 1. Human leukocyte antigen (HLA) typing revealed HLA-DQB1*05:01 and HLA-DRB1*10:01 alleles. The patient was followed up for nearly 6 months and showed no recurrent pain or sleep disruptions. The clinical course and treatment schedule are shown in Fig. 2.

## Discussion and conclusions

Sleep disorder is the most prominent symptom of anti-IgLON5 antibody-related encephalopathy [1]. However, in this case, although there was a sleep disorder, it was not very obvious. Unfortunately, polysomnography was not performed due to the limitation of hospital medical conditions. A simple portable sleep monitoring device was used to monitor the patient, including the nasal airflow, chest and abdominal breathing, blood oxygen saturation, and electrocardiogram parameters. The results showed that the patients had no obvious hypoxemia and sleep apnoea. This patient’s signs and symptoms were consistent with ‘cognitive impairment associated with chorea’ [2]. However, the clinical presentation was mild, and his condition rapidly improved. This may explain why the IgLON5 antibody was positive only in the serum, and the titre was low.

Immunotherapy is the primary treatment for autoimmune encephalopathy; however, its efficacy remains uncertain. Our patient had a fever and abnormal infection indices. Despite two negative viral antibody tests, the possibility of viral infection was still considered. On initiating antiviral treatment, his clinical symptoms and laboratory results improved promptly and dramatically, and the titre of serum IgLON5 antibody also decreased. Based on clinical practice, we presume that IgLON5 antibody may be produced in viral infections in genetically susceptible individuals and may regress spontaneously with the resolution of viral encephalitis. Recent studies have shown that viral encephalitis (VE) can cause autoimmune encephalitis (AE). Among them, HSV encephalitis can cause anti-NMDAR encephalitis, which has been confirmed by many studies [3, 4]. There are mainly three hypotheses about anti-NMDAR encephalitis induced by HSV encephalitis [5, 6]: molecular simulation, virus infection leading to inflammatory damage of the blood-brain barrier and neuronal damage, and necrosis, which leads to exposure and release of antigen determinants as autoimmune targets. Inflammation of the central nervous system in the course of infection causes immune activation. We speculate that the relationship between virus infection and anti-IgLON5 encephalopathy may also be related to the above hypothesis.

In this report, we discussed the aetiology, clinical manifestations, treatment, and prognosis of IgLON5 antibody-related encephalopathy and proposed a pathophysiological hypothesis. Infection and genetic predisposition may be important disease contributors, and patients with a mild disease may demonstrate a better prognosis.

## Data Availability

All data generated or analysed during this study are included in this published article***.***

## Abbreviations

CRP: C-reactive protein
CSF: Cerebrospinal fluid;
ESR: Erythrocyte sedimentation rate
HLA: Human leukocyte antigen
PCT: Procalcitonin
VE: Viral encephalitis
AE: Autoimmune encephalitis
